# Hallmarks and mechanisms of cellular senescence in aging and disease

**DOI:** 10.1038/s41420-025-02655-x

**Published:** 2025-08-04

**Authors:** Amir Ajoolabady, Domenico Pratico, Suhad Bahijri, Basmah Eldakhakhny, Jaakko Tuomilehto, Feng Wu, Jun Ren

**Affiliations:** 1 National Clinical Research Center for Interventional Medicine, Shanghai, 200032 China; 2 State Key Laboratory of Cardiovascular Diseases, Zhongshan Hospital, Fudan University Shanghai, 200032 China; 3 Alzheimer’s Center at Temple, Lewis Katz School of Medicine, Temple University, Philadelphia, PA 19140 USA; 4 Department of Clinical Biochemistry, Faculty of Medicine, King Abdulaziz University, Jeddah, Saudi Arabia; 5 Saudi Diabetes Study Research Group, King Fahd Medical Research Centre, King Abdulaziz University, Jeddah, Saudi Arabia; 6 Food Nutrition and Lifestyle Research Unit, King Fahd Medical Research Centre, King Abdulaziz University, Jeddah, Saudi Arabia; 7 Department of Public Health, University of Helsinki, 00014 Helsinki, Finland; 8 Department of Cardiology, The 72nd Group Army Hospital, Huzhou University, Huzhou, Zhejiang 313000 China; 9 Shanghai Institute of Cardiovascular Diseases, Department of Cardiology, Zhongshan Hospital Fudan University, Shanghai, 200032 China

**Keywords:** Senescence, Diseases

## Abstract

Cellular senescence, often referred to simply as “senescence”, is a complex intracellular process with diverse biological, physiological, and pathological roles. Biologically, it is essential for embryogenesis and development. Physiologically, senescence acts as a safeguard against tumorigenesis by preventing the proliferation of damaged or defective cells. However, persistent activation of senescence can contribute to various pathological conditions, particularly those associated with aging, cancer, and other chronic diseases such as liver and pulmonary diseases. Growing evidence links aging to heightened activation of cellular senescence, leading to the accumulation of senescent cells. Here in this perspective, we aim to decipher the latest molecular mechanisms and regulatory pathways of cellular senescence in the context of aging and aging-related diseases. Additionally, we discuss emerging research directions, highlighting current limitations and gaps in the field. Addressing these challenges may not only advance our understanding of senescence but also uncover new therapeutic opportunities.

## FACTS

- Aging is linked with cellular senescence and subsequent accumulation of senescent cells in various organs, ultimately, contributing to the pathogenesis and progression of multiple aging-associated disorders.
- Aging triggers cellular senescence through multiple mechanisms such as DNA damage and oxidative stress.
- In contrast to what initially believed to be, it is now evident that the link between aging and cellular senescence is far more complex

## Introduction: a glimpse at cellular senescence

**Fig. 1:**
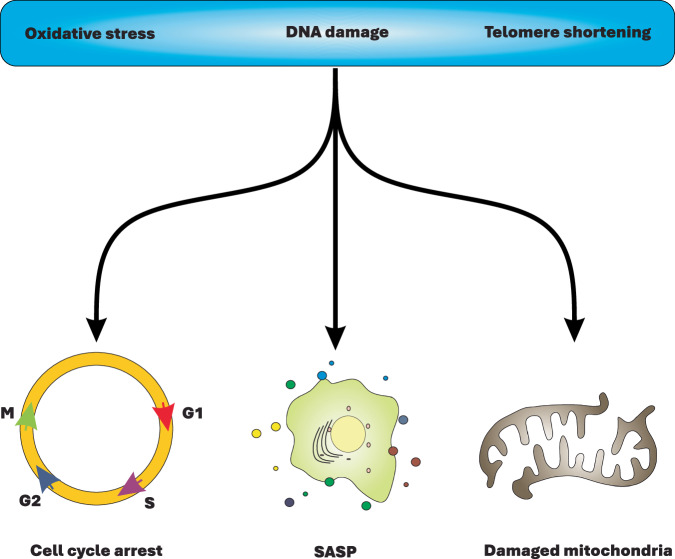
The hallmarks of cellular senescence. DNA damage, oxidative stress, and telomere shortening are the primary triggers of cellular senescence, endowing senescent cells with deregulated metabolism and mitochondrial damage, SASP, and arrested cell cycle.

Cellular senescence is an evolutionarily conserved process that leads to an irreversible arrest of the cell cycle, occurring more prominently in eukaryotes than in prokaryotes [1–4]. It is triggered by various forms of cellular stressors, including DNA damage, telomere shortening, oxidative stress, and oncogene activation, in an effort to halt the proliferation of defective cells. This serves to prevent malignant transformation or switch to apoptosis [2, 5–8]. In this context, cellular senescence can be viewed as a primary anti-apoptotic and anti-tumorigenic mechanism. However, accumulative evidence suggests that persistent activation of cellular senescence can contribute to pathological conditions, including tumorigenesis and progression of diseases such as liver, skin, and pulmonary disorders [9–11]. Morphologically, senescent cells are characterized by a flattened shape and enlarged size. More notably, they secret a variety of cytokines, chemokines, growth factor, immune modulators, proteases, and matrix metalloproteinases (MMPs), collectively known as the “senescence-associated secretary phenotype (SASP)” [12–14]. It is proposed that SASP plays a cardinal role in amplifying the detrimental effects of cellular senescence on disease progression [15, 16]. Moreover, deregulated metabolism, mitochondrial/lysosomal dysfunction, and macromolecular damage are among other hallmarks associated with senescent cells (Fig. 1) [1, 3].

## Aging and mechanisms of cellular senescence

**Fig. 2:**
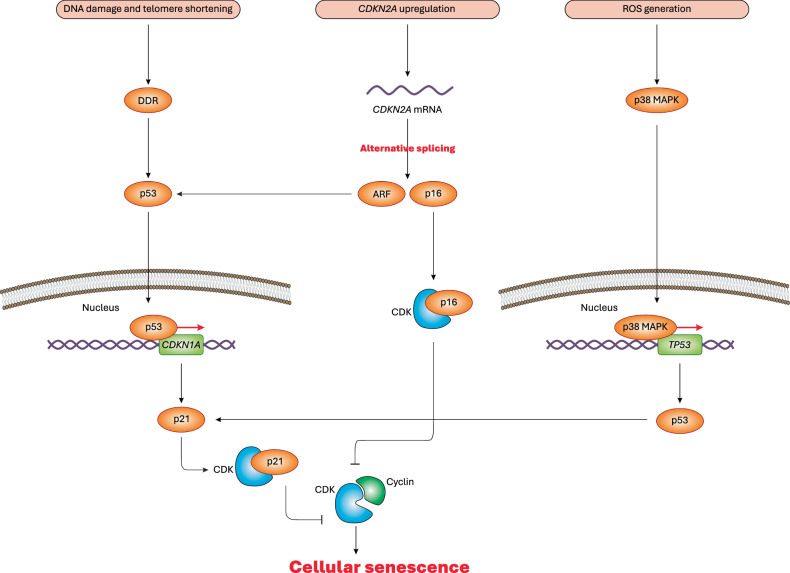
Molecular mechanisms of cellular senescence. DNA damage and telomere shortening trigger DNA damage response (DDR), leading to activation of p53 transcription factor, which translocates to the nucleus causing transcriptional activation of *CDKN1A*. As a result, p21 specifically binds to CDKs and blocks their complex formation with cyclins, ultimately, resulting in cell cycle arrest and cellular senescence. Aging-associated ROS generation also activates the p38 MAPK signaling, leading to transcriptional activation of *TP53*, which encodes p53, thereby activating the p21-CDK axis, thus inhibiting CDK-cyclin complexes, ultimately, culminating in cell cycle arrest and cellular senescence. Last but not least, aging-associated *CDKN2A* upregulation can lead to alternative splicing of its mRNA, which produces ARF and p16 tumor suppressor proteins. ARF preserves p53 thus inducing the p53-p21-CDK axis, thereby blocking the cell cycle and inducing cellular senescence. Besides, p16 is another key CDKi that can bind and inhibit specific CDKs, thereby blocking the cyclin-CDK complexes, ultimately, igniting cellular senescence.

One of the hallmarks of biological aging is the accumulation of senescent cells [17, 18]. Aged cells are thought to be more prone to senescence than young cells [18]. The connection between aging and cellular senescence stems from aging-induced disruptions in cellular processes and homeostasis [19–21]. For instance, aging promotes DNA damage and telomere shortening, which, in turn, activates the DNA damage response [22, 23]. The *TP53* gene encodes the p53 tumor suppressor protein, a transcription factor that regulates cell division, prevents uncontrolled proliferation, and suppresses tumorigenesis [24–26]. Aging-induced DNA damage response activates p53, which in turn transcriptionally regulates key genes involved in cellular senescence [27]. One such target is *CDKN1A* (cyclin dependent kinase inhibitor 1 A), which encodes p21 protein, a cyclin-dependent kinase inhibitor (CDKi) [28, 29]. p21 inhibits cyclin-dependent kinases (CDKs), including CDK1, CDK2, CDK4, CDK6, by preventing their association with cyclins. This inhibition halts cell cycle progression at the G1 and S phases (Fig. 2) [28, 30, 31]. Mechanistically, cyclins bind to inactive CDKs to form active CDK-cyclin complexes, which drive cell cycle progression through phosphorylation of downstream targets [32]. However, p21 disrupts this process, reinforcing cellular senescence as a response to aging.

Additionally, aging is known to upregulate *CDKN2A* (cyclin-dependent kinase inhibitor 2 A) [33]. This gene encodes p16, a tumor suppressor protein and an alternative CDKi that binds to CDK4 and CDK6, preventing the formation of CDK4/6-cyclin complexes. This inhibition ultimately leads to cell cycle arrest and cellular senescence [34, 35]. Furthermore, alternative splicing of *CDKN2A* mRNA produces another distinct tumor suppressor protein, ARF [36]. Mechanistically, ARF inhibits MDM2 (mouse double minute 2 homolog), an E3 ubiquitin-protein ligase, thereby preventing MDM2-mediated degradation of p53. This stabilization of p53 activates the p53-p21-CDK axis, leading to cell cycle arrest and the induction of cellular senescence (Fig. 2) [34, 35].

During aging process, mitochondrial ATP production declines, leading to increased reactive oxygen species (ROS) generation and heightened oxidative stress [37]. Additionally, aging weakens cellular antioxidant defense system, further exacerbating oxidative stress [37, 38]. Persistent oxidative stress can cause significant damage to organelles, membranes, macromolecules, and can even lead to DNA damage [37, 39]. It is hypothesized that aging-induced oxidative stress triggers DNA damage, leading to activation of the p53-p21-CDK axis and subsequent cellular senescence [40–42]. This is why administration of antioxidants can block or delay the onset of cellular senescence [40–42]. In an alternative mechanism, oxidative stress and elevated ROS levels can activate p38 MAPK (mitogen-activated protein kinase), triggering a signaling cascade that upregulates the transcription of *TP53*/p53. As discussed above, this activates the p53-p21-CDK signaling, thereby inhibiting the CDK-cyclin complexes, and inducing cellular senescence (Fig. 2) [43–45].

It is important to note that, beyond the mechanisms discussed, activation and regulation of cellular senescence may involve other pathways depending on disease models and cell types. Several comprehensive reviews have extensively explored alternative mechanisms that could drive cellular senescence in mammalian cells [46–48]. Indeed, the complex interplay among aging, cellular senescence, and associated diseases is driven by a cascade of intricate molecular mechanisms and signaling pathways. In the following sections, we will explore these mechanisms in depth, highlighting the latest findings in the field.

## Aging-associated cellular senescence and the METTL1-WDR4 complex

Transfer RNAs (tRNAs) are small RNAs that participate in mRNA translation and protein synthesis. Mechanistically, tRNAs transfer amino acids to the growing amino acid chain in ribosomes corresponding to mRNA codons [49, 50]. Post-translational chemical modification of tRNAs regulate/modulate their function; therefore, affecting mRNA translation [51–53]. In eukaryotes, METTL1 (methyltransferase 1, tRNA methylguanosine) enzyme catalyzes N7-methylguanosine methylation (m7G) of tRNA in internal sites [54, 55]. WDR4 (WD repeat domain 4) enzyme also acts as a cofactor and forms a complex with METTL1 [56, 57].

A recent mouse study revealed that activation of the METTL1-WDR4 complex induces m7G methylation at guanosine 46 (m7G46) of tRNA, thereby influencing mRNA translation and promoting cellular senescence [58]. The study demonstrated that aging-associated cellular senescence disrupted the METTL1-WDR4 complex and reduced tRNA m7G46 levels, leading to rapid tRNA degradation (RTD) [58]. This disruption caused ribosomal dysfunction in translating specific mRNA codons, thereby impairing mRNA translation and synthesis of essential proteins involved in cellular function, such as ribosomal biogenesis and activation of signaling pathways like Wnt signaling [58]. Furthermore, ribosomal dysfunction induces both integrative and ribotoxic stress responses, resulting in an increase in SASP [58]. Conversely, eEF1A (eukaryotic elongation factor 1 A), a key binding protein that delivers amino-acylated tRNAs (aa-tRNAs) to ribosomes during mRNA translation [59], plays a protective role. Transgenic activation of eEF1A was found to reverse RTD and mitigate SASP effects [58], highlighting its potential in counteracting aging-related cellular dysfunction.

Collectively, these findings suggest that aging-induced cellular senescence may deactivate the METTL1-WDR4 complex and therefore reduce m7G46 modification of tRNAs, which impairs mRNA translation of certain proteins, ultimately, conferring senescent phenotypes such as increased SASP. Based on these findings, we hypothesize that cellular senescence weakens the METTL1-WDR4 complex and eEF1A, potentially as a mechanism to suppress ribosomal activity and protein synthesis, thereby halting specific cellular processes while sustained or exacerbating SASP. However, a critical limitation of this study is the lack of an in-depth investigation into the molecular mechanisms underlying senescence-driven inhibition/disruption of the METTL1-WDR4 complex and eEF1A. Future research should aim to elucidate these mechanisms in detail, identifying key intermediary molecules linking cellular senescence to METTL1-WDR4 dysfunction. These could serve as potential therapeutic targets to counteract the effects of cellular senescence-induced ribosomal dysfunction and impaired protein translation in the context of aging and associated diseases, highlighting the study limitations in translational and therapeutic aspects [8]. Additionally, the study did not explore the functional consequences of altered Wnt signaling on intracellular processes related to cellular senescence and SASP. Further fundamental studies are needed to dissect the molecular events downstream of Wnt signaling alterations and their broader impacts on cellular function, SASP regulation, and gene expression modifications.

However, in conditions where METTL1 is deactivated, reactivation of eEF1A could serve as a potential strategy to delay cellular senescence by enhancing tRNA deliverance and ribosomal elongation. Despite these significant findings, the connection between aging, cellular senescence, and tRNA m7G46 modification remains largely unexplored, underscoring the need for further basic studies with a specific focus on molecular mechanisms, protein-protein interactions, and the intermediate molecules linking cellular senescence with intracellular events of protein translation. Moreover, this highlights the importance of employing biochemical techniques to uncover intracellular interactions between senescence-associated proteins and mRNA/protein translation factors. Additionally, advanced molecular biology techniques should be utilized to investigate gene expression modifications that potentially govern the interplay between cellular senescence and the METTL1-WDR4 axis.

## Aging-associated cellular senescence and the STAT6 signaling

**Fig. 3:**
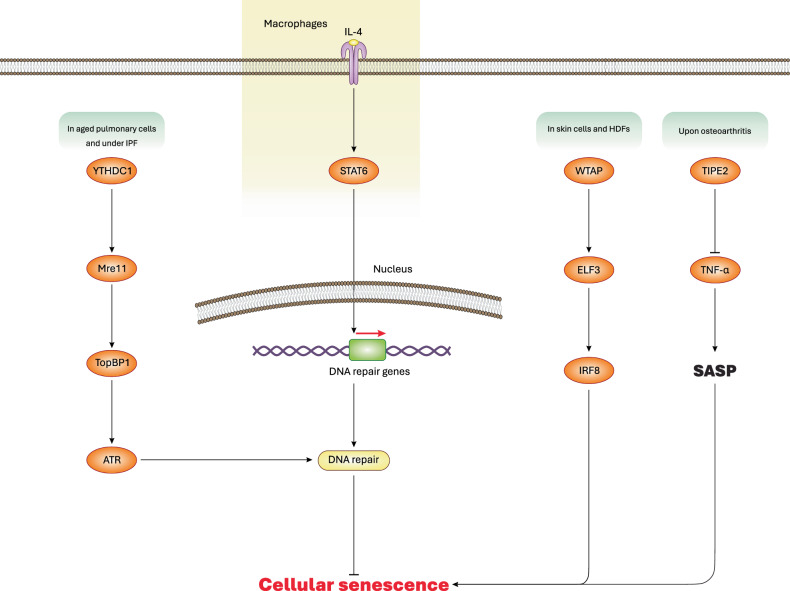
Specific mechanisms and regulation of cellular senescence. Activation of YTHDC1 promotes the interaction between Mre11 and TopBP1 proteins, thereby activating ATR molecule, which promotes DNA repair thus preventing cellular senescence. Besides, activation of the IL-4-STAT6 signaling can transcriptionally upregulate DNA repair genes, leading to improved DNA homeostasis, averted DNA damage, and inhibition of cellular senescence. WTAP also function as a subunit of m6A methyltransferase complex, causing activation of the ELF3-IRF8 axis, resulting in cellular senescence. Conversely, activation of TIPE2 antagonizes TNF-α secretion and SASP, thereby alleviating or reversing cellular senescence.

Aging is associated with dysfunction of the immune system, termed “immunosenescence” [60]. Immunosenescence differs from cellular senescence. Immunosenescence is the consequence of aging on immune cells characterized by reduced proliferation and dysfunction, while cellular senescence denotes a permanent halt on cell growth and proliferation, occurring in all cell types including immune cells [60, 61]. To this end, cellular senescence aims to prevent the division of defective cells such as those bearing unresolvable DNA damage [60, 61]. Type 2 cytokines including IL-4 (interleukin-4), IL-5, IL-9, and IL-13 mediate type 2 immune responses [62]. In aged mice, deficiency of type 2 cytokines and absence of their signaling induced organismal aging and cellular senescence in macrophages due to accumulative DNA damage [63]. However, restoring type 2 cytokines (e.g. IL-4) reactivated STAT6 (signal transducer and activator of transcription 6) signaling, thereby delaying macrophage senescence through transcriptional activation of DNA repair genes including those involved in Fanconi anemia DNA repair disorder or in homologous recombination-mediated DNA repair [63]. Indeed, *STAT6* deficiency was shown to provoke DNA release into the cytosol (indicative of DNA damage), thereby leading to activation of cellular senescence. However, restoring IL-4 signaling reversed cellular senescence in macrophages and augmented healthy life span in aged mice. Taken together, these data suggest that activation of the IL-4-STAT6 axis by type 2 cytokines inhibits macrophage senescence owing to improved DNA repair mechanisms (Fig. 3). Moreover, these findings suggest that STAT6 primarily functions as a transcription factor regulating the expression of DNA repair genes. However, this study did not provide a detailed elucidation of STAT6 signaling, its downstream pathways, and its role in transcriptional regulation of DNA repair genes, enhanced DNA repair mechanisms, and ultimately, delayed cellular senescence in macrophages. Further studies should further investigate STAT6-regulated transcription of DNA repair genes and their downstream effects on DNA repair mechanisms and cellular senescence inhibition. Only then can we take a step toward translating these findings [63] into practical therapeutic strategies targeting STAT6-regulated macrophage senescence, particularly, in diseases closely linked to macrophage aging. Additionally, given that type 2 cytokines act on diverse cell types, findings of this study [63] should be validated in both immune and non-immune cells beyond macrophages. This would enhance the accuracy, translational merits, and applicability of the results to a broader range of senescent cells and aging-associated immune/non-immune disorders. Furthermore, a critical oversight of this study was the conflation of immunosenescence with cellular senescence, which are distinct biological processes. Such misinterpretations underscore the need for appropriate models and quantitative/qualitative techniques to accurately differentiate immunosenescent cells from senescent cells and SASP-associated phenotypes [64]. Interestingly, this study, perhaps for the first time, endorsed therapeutic and anti-senescence/senomorphic potential of type 2 cytokines in aging and senescence-associated diseases. Senomorphics, defined as drugs that slow down senescence or alleviate SASP [65, 66], represent a promising avenue for intervention. However, this concept must be rigorously examined in both preclinical and clinical settings before type 2 cytokines can be considered reliable senomorphic agents for treating aging-associated diseases.

## Cellular senescence and SASP regulation by TIPE2

Aging heightens the risk of a degenerative join disease, osteoarthritis [67–69]. Increased expression of *TNF*/TNF-α (tumor necrosis factor) is a predominant feature of osteoarthritis, causing inflammation and joint pain in patients [70–73]. Therefore, it is perceived that TNF-α inhibition may alleviate symptoms of this aging-associated disease. In addition, inhibiting TNF-α using monoclonal antibodies does not elicit complete inhibition of TNF-α and often demands several doses of treatments [74–76]. On the other hand, TIPE2 (TNF alpha induced protein 8 like 2) is a protein that negatively regulates innate and adaptive immunity and inflammation [77].

In a recent study using mouse model of accelerated aging (*Zmpste24*^*−/−*^), adenoviral transfection of *TIPE2* gene led to increased intensity of safranin O staining (orange-red color containing glycosaminoglycan content) in knee’s articular cartilage (AC) region compared with control mice [78]. In *Zmpste24*^*−/−*^ mice, chondrocytes, a key cartilage cell type, exhibited senescence phenotypes. *TIPE2* transfection was shown to suppress TNF-α secretion, β-galactosidase activity, and *CDKN2A*/p16 expression, thereby delaying cellular senescence and inflammation [78, 79]. Collectively, these findings suggest that inducible activation of TIPE2 can reduce or inhibit TNF-α secretion, suppress SASP and inflammation, and ultimately alleviate cellular senescence and osteoarthritis (Fig. 3) [78]. However, despite these important insights, this study lacks an in-depth exploration of the molecular mechanisms underlying TIPE2-mediated regulation of TNF-α expression/secretion and cellular senescence. Additionally, it does not provide mechanistic insights into how cellular senescence contributes to osteoarthritis development. Addressing these gaps will require comprehensive basic research focusing on molecular mechanisms, protein interactions, and gene expression analyses. Future studies should aim to identify missing regulatory pathways, which could lead to translational advancements. Pending these investigations, further preclinical and clinical studies will be necessary to evaluate whether TIPE2 can be considered a potential senomorphic for osteoarthritis treatment.

## Regulation of cellular senescence by WTAP-mediated m6A modification

N6-methyladenosine (m6A) is a common type of post-transcriptional modification in RNAs that regulate cell function and implicated in various diseases [80–82]. Both human and mouse models have demonstrated that aging is associated with heightened m6A modification of RNAs (such as in aged brain) [83–86].

In a recent mouse study, proteomics analysis (involving systematic quantification and identification of the proteome in a biological sample) identified *WTAP*/WTAP (Wilms’ tumor 1-associating protein) and its expression to be significantly involved in senescence of human dermal fibroblasts (HDFs) and skin tissues [87]. Consistently, aged models of HDFs and skin cells exhibited increased expression of *WTAP*/WTAP, while the genetic ablation of *WTAP*/WTAP prevented cellular senescence [87]. Functionally, WTAP is an essential component of the m6A methyltransferase complex, directing the complex to specific target mRNAs [88]. In this study, WTAP was shown to bind to *ELF3* (E74 like ETS transcription factor 3) mRNA, mediating its m6A modification and enhancing its expression [87]. As a result, the ELF3 transcription factor was activated, leading to an increase in its protein levels and its subsequent binding to the *IRF8* (interferon regulatory factor 8) promoter region, thereby upregulating *IRF8* transcription and protein level. Ultimately, IRF8 induced cellular senescence and provoked SASP of senescent HDFs and skin cells, promoting skin aging in vivo [87].

These findings underscore the pivotal role of WTAP-mediated m6A modification of *ELF3 mRNA* and the subsequent activation of IRF8 protein in driving aging and senescence of HDFs and skin cells (Fig. 3). Despite the valuable contributions of this study [87], it lacks a thorough investigation into the downstream molecular mechanisms linking IRF8 activation to cellular senescence induction. Addressing this gap requires additional basic studies to elucidate these molecular connections, potentially paving the way for therapeutic strategies, such as targeted inhibition of IRF8-mediated cellular senescence to combat skin aging. Such endeavors also require exploration and affirmation by clinical studies. Broadly, therapeutic approaches could focus on targeting the WTAP-ELF3-IRF8 axis and manipulating m6A modifications in relevant RNAs to inhibit cellular senescence and associated aging-related diseases. However, as previously discussed, current experiments and clinical evidence remains insufficient to support such interventions. Additionally, a crucial yet underexplored question is what molecular and mechanistic events follow cellular senescence in skin cells, and how they contribute to skin aging. The original study [87] primarily attributed IRF8-mediated senescence to SASP as the key driving force of skin aging, but this explanation remains simplistic. Further molecular investigations and basic studies are needed to uncover additional mechanisms linking cellular senescence to accelerated skin aging, potentially identifying novel therapeutic targets for intervention.

## The role of YTHDC1 in aging-associated cellular senescence

Idiopathic pulmonary fibrosis (IPF) is a lung disease featured by interstitial fibrosis, termed “idiopathic” due to the lack of obvious causes [89–91]. Accumulative evidence suggests that advanced aging is closely linked to the etiology of IPF [92–94]. In one mechanism, aging induces severe pulmonary DNA damage [95–97], a profound driver of cellular senescence [98–100], thus exacerbating IPF [101–103]. Hence, the pathophysiology of pulmonary fibrosis and IPF are closely associated with cellular senescence.

In a recent study, *YTHDC1*/YTHDC1 (YTH N6-methyladenosine RNA binding protein C1) was identified as binding to m6A on RNAs, and is predominantly expressed in ATII (pulmonary alveolar epithelial type 2) cells [104, 105]. *YTHDC1* expression was found to be downregulated in ATII cells during pulmonary fibrosis [101]. Examination of mouse ATII cells revealed that enforced overexpression of *Ythdc1* prevented cellular senescence and fibrosis, independent of YTHDC1’s primary role in m6A-binding, while *Ythdc1* deficiency accelerated IPF development in mice [101]. Mechanistically, YTHDC1 mediated the interaction between Mre11 and TopBP1 (MRE11 homolog, double strand break repair nuclease/DNA topoisomerase II binding protein 1), which led to activation of ATR (ataxia telangiectasia-mutated and RAD3-related), a molecule involved in promoting DNA repair, thereby preventing cellular senescence and fibrosis [101]. Of note, the primary role of Mre11 is to process and monitor double-strand DNA breaks [106], while TopBP1 acts as a scaffold that recruits proteins involved in DNA repair [107]. ATR accumulates at DNA breaks, guiding and regulating DNA repair by phosphorylating downstream targets such as Chk1 (checkpoint kinase 1) [108]. Therefore, activation of the YTHDC1-Mre11-TopBP1-ATR signaling significantly facilitates DNA repair. For more details on DNA repair mechanisms, several comprehensive reviews are available in the literature [109–111]. Taken together, these data indicate that YTHDC1 stimulates DNA repair response and prevents senescence in aged pulmonary cells (Fig. 3). Hence, upregulation of *YTHDC1*/YTHDC1 in pulmonary cells could be a potential therapeutic strategy for IPF, although further studies are necessary to confirm its safety and clinical applicability.

Although cellular senescence is primarily associated with permanent cell cycle arrest, it also plays a crucial role in elucidating the molecular mechanisms linking cellular senescence to the pathogenesis of pulmonary fibrosis and IPF. However, the abovementioned study by *Zhang* and colleagues [101] largely overlooked this aspect. A key limitation of their research is that, while they demonstrated a link between cellular senescence and increased fibrosis, they did not deeply explore the underpinning molecular mechanisms. This highlights the need for future basic studies to investigate how cellular senescence drives fibrosis, both generally or in the specific context of IPF. To achieve this, research should integrate traditional imaging techniques [112] with cutting-edge cellular and molecular technologies, including omics approaches such as genomics, transcriptomics, proteomics, and metabolomics [113]. These techniques remain underutilized in the field, likely due to inadequate experimental design and research roadmap. Existing evidence indicates that senescent cells contribute to fibrosis by secreting chemokines, cytokines, and growth factors through SASP [114]. In particular, senescent ATII cells release abundant SASP factors, which induce fibrosis in neighboring fibroblasts and alveolar macrophages, further exacerbating pulmonary fibrosis [115–117]. Given these insights, further research should focus on unraveling the molecular mechanisms by which cellular senescence impacts IPF and pulmonary fibrosis, ultimately guiding targeted therapeutic strategies.

## Conclusions and final remarks

In summary, accumulating evidence suggests that aging is closely associated with induction of cellular senescence and subsequent accumulation of senescent cells in various organs, ultimately, contributing to the pathogenesis and progression of multiple aging-associated disorders. Simply put, aging primarily induces cellular senescence through mechanisms such as oxidative stress and DNA damage. However, when examining different disease models and cell types, it becomes evident that the relationship between aging and cellular senescence is far more complex than it initially appears. Recent studies have provided valuable insights into the molecular mechanisms governing cellular senescence and its intricate regulation across various diseases and experimental models. However, this perspective offers only a glimpse into this multifaceted process, and further research is necessary for a comprehensive understanding. Basic studies are particular essential to deepen our knowledge of cellular senescence, including its mechanisms, regulation, and connection to aging-related diseases. At its core, cellular senescence functions as a “stopwatch” that halts the cell cycle, preventing cell proliferation and growth under conditions of cellular stress, including DNA damage, telomere shortening, and oxidative stress. Mechanistically, the proves is driven by the activation of the p53 tumor suppressor, which transcriptionally upregulates *CDKN1A* encoding p21, a CDKi. This, in turn, inhibits the formation of cyclin-CDK complexes, leading to cell cycle arrest and senescence. Although SASP has been widely recognized as a key driver of the pathological effects of senescent cells, other yet unidentified mechanisms may also contribute to the disease-associated roles of cellular senescence. As research progresses, these mechanisms continue to be uncovered and this trend is expected to persist. However, as discussed in detail in the studies above, the therapeutic and translational potential of current findings remains limited or requires additional preclinical and clinical studies. To effectively target cellular senescence in aging-associated diseases, future research must employ both traditional and cutting-edge technologies, ensuring a comprehensive and integrative approach in the foreseeable future.
